# Short- and long-term survival after open versus endovascular repair of abdominal aortic aneurysm—Polish population analysis

**DOI:** 10.1371/journal.pone.0198966

**Published:** 2018-06-14

**Authors:** Bartosz Symonides, Andrzej Śliwczyński, Zbigniew Gałązka, Jarosław Pinkas, Zbigniew Gaciong

**Affiliations:** 1 Department of Internal Medicine, Hypertension and Vascular Diseases, The Medical University of Warsaw, Warsaw, Poland; 2 Department of Analysis and Strategy, The National Health Fund, Warsaw, Poland; 3 Department of Vascular and Endocrine Surgery, The Medical University of Warsaw, Warsaw, Poland; 4 Department of Healthcare Organizations and Medical Jurisprudence, Centre of Postgraduate Medical Education, Warsaw, Poland; Universidad Francisco de Vitoria, SPAIN

## Abstract

**Objectives:**

The aim of the study was to compare short and long-term mortality and readmissions in patients with non-ruptured abdominal aortic aneurysm (AAA) treated with endovascular aortic repair (EVAR) or open aneurysm repair (OAR).

**Design:**

Retrospective survival analysis based on prospectively collected medical records of the national Polish public health insurer.

**Materials:**

In the National Health Fund database we identified all patients who underwent elective open or endovascular treatment of AAA between January 1^st^ 2011 and March 22^nd^ 2016. The data on mortality, selected concomitant diseases and readmissions were collected. A total of 7805 patients (mean age 70.9±8.1 yrs, 85.8% males) underwent OAR (n = 2336) or EVAR (n = 5469). A median follow up was 27.5 months (IQR range 10.0–38.4 months).

**Methods:**

The primary outcome variable was all-cause mortality, secondary outcomes included 30-day mortality and readmissions. Kaplan–Meier (K-M), Cox proportional-hazards and propensity score analyses were performed for primary and secondary outcomes adjusting for repair type of AAA (OAR vs. EVAR), age, sex and concomitant diseases.

**Results:**

EVAR patients had higher all-cause mortality (6.4% vs. 4.6% P = 0.002, adjHR 1.34, 95%CI 1.07–1.67, P = 0.010) compared with OAR. The mortality risks for OAR patients decreased below those for EVAR patients after 9.9 months. Of all the tested confounding factors only age independently and significantly influenced long-term mortality. Readmissions occurred more often in EVAR than in OAR (16.5% vs. 8.4% P<0.001, adjHR 2.15, 95%CI 1.84–2.52, P<0.001) independently from other covariants. Survival and readmissions Kaplan-Meier curves remained statistically different between OAR and EVAR patients after propensity score matching.

**Conclusions:**

Survival benefit of EVAR over OAR disappeared early during the first year after procedure, particularly in patients below 70 years of age, accompanied by an increased frequency of readmissions of EVAR patients. Our data suggest re-evaluation of the strategy for AAA management in vascular units in the country.

## Introduction

Abdominal aortic aneurysm (AAA) is a common disease in the Western population, with a prevalence of 2% to 5% in men ≥ 65 years of age, with very high mortality rates related with AAA rupture [1]. Recent Polish epidemiological studies revealed the prevalence of abdominal aortic aneurysm similar to other European countries [2]. Endovascular repair of abdominal aneurysm (EVAR) has become the method of choice over open aneurysm repair (OAR) due to lower perioperative mortality found in randomized clinical trials (RCTs) [3,4]. However data from these trials revealed that survival advantage of EVAR disappears during long-term follow-up [5,6]. In the DREAM study two years after randomization the cumulative survival rates were 89.6% for OAR and 89.7% for EVAR. The advantage of EVAR over OAR regarding aneurysm-related death (5.7% vs. 2.1%) was entirely accounted for by events occurring in the perioperative period, with no significant difference in subsequent aneurysm-related mortality [5]. In EVAR-1 study 4 years after randomization, all-cause mortality was similar in the two groups (about 28%), despite the fact that there was a persistent reduction in aneurysm-related deaths in the EVAR group (4% vs. 7%, p = 0.04). The proportion of patients with postoperative complications within 4 years of randomization was 41% in the EVAR group and 9% in the OAR group [6].

Although RCTs are considered the "golden standard" for comparing medical procedures they may not reflect every-day practice of AAA at population level so the applicability of their results in clinical practice may be limited [7]. Patients treated in with EVAR or OAR in the clinical trial setting were preselected according to strict inclusion/exclusion criteria and therefore they have on average fewer and less severe comorbidities and are more likely to be male than patients encountered in clinical practice thus representing lower-risk patients [7,8].

It is of note that patients with AAA in RCTs must be good candidates for both procedures, so the method of treatment might be suboptimal for the specific patient. RCTs are usually carried out in high-volume centers employing vascular surgeons experienced in endovascular technique and equipped with the best hardware. Of 4799 patients assessed for eligibility into the EVAR-1 study as many as 1795 were judged unsuitable for EVAR device, 457 were unfit for open repair and finally only 1082 (22.5%) were randomized [6].

The results obtained from national registries may be easier to generalize than the results from RCTs as they reflect every-day practice and not just the practice of centers of excellence [9]. However registries are carried out in only in a limited number of countries, and may be biased due to non-inclusion of all AAA repairs.

Moreover, management and results of treatment of AAA varies between countries [1,9,10]. The differences include mean age at the time of intact AAA repair (from 68.9 years in Hungary to 74.6 years in Australia), mean diameter of aorta (5.9 cm in Australia and the United States to 6.5 cm in Finland), different EVAR usage (from 27.8% in Hungary to 79.5% in United States) [1]. During the period from 2005 through 2012 relative aneurysm repair rate was twice less common in England than in the United States but aneurysm rupture occurred twice more frequently in England and aneurysm-related death was over three times more common than in the United States [10].

The long-term survival and outcomes of EVAR and OAR have rarely been studied on a population level, and they either included selected age groups [7] or were limited to a part of the country [8].

In Poland EVAR is reimbursed on a regular basis by the NHF since 2009 and the data regarding medical procedures is available in digital form [11].

The aim of the study was to compare short and long-term mortality and readmissions in patients with non-ruptured abdominal aortic aneurysm treated with EVAR vs. OAR using population data from the entire country.

## Materials and methods

We used data collected by the National Health Fund (NHF), the only public and obligatory health insurer in Poland. In case of medical procedures related to the treatment of AAA, the NHF is practically the single payer that signs contracts with public and private healthcare providers.

The NHF database tracks all patients admissions, main diagnoses, concomitant diseases and medical procedures longitudinally throughout the entire country. Additionally, the database contains information on birth and death dates. The database search included the period from Jan 1^st^ 2011 to March 22^nd^ 2016. Inclusion criteria were patients with International Classification of Diseases Tenth Revision (ICD-10) diagnosis code of I71.4 (abdominal aortic aneurysm, without rupture), who underwent either open or endovascular treatment of AAA with International Classification of Diseases Ninth Revision procedure (ICD-9) codes of 38.424 for OAR or 39.711 for EVAR (S1 Table). The earliest procedure was considered the index one. Exclusion criteria included ICD-10 codes for ruptured or extended AAA to thoracic part of aorta (S1 Table). No other inclusion or exclusion criteria were applied including patients specific (e.g. diameter of AAA) or procedure specific (type of stentgraft used). All patients fulfilling inclusion and exclusion criteria who underwent the index procedure within the time period of interest were included in the study disregarding duration of follow-up.

The database was also searched for selected concomitant diseases (hypertension, chronic renal failure, coronary artery disease, diabetes mellitus, stroke recorded before or at the time of index procedure according to specific ICD-10 codes (S2 Table).

All data released from NHF were fully anonymized by applying encrypted personal identifiers before the authors had any access to them.

The primary outcome variable was all-cause mortality, secondary outcomes included 30-day mortality and readmissions. Readmission as secondary outcome was defined as the first all-cause re-hospitalization to vascular unit (according to NHF specific codes).

Survival analysis was performed for primary and secondary outcomes adjusting for repair type of AAA (OAR vs. EVAR), age, sex and concomitant diseases.

All-cause mortality outcomes were censored at the end of the study on March 22^nd^ 2016, 30-day all-cause mortality at 30 days. Readmissions were censored at death or at the end of the study.

### Statistics

Variables were compared using Fisher’s exact test and Mann–Whitney U test. Estimates of cumulative event rates were calculated by means of the Kaplan–Meier method with the log-rank comparison of survival curves. Cox proportional-hazards analyses were performed for primary and secondary outcomes controlling for repair type (OAR vs. EVAR), age, sex, concomitant diseases and readmissions (expressed as total number of events). Analysis were also performed in age subgroups in patients above 70 years (70+) or younger (70-). Propensity score analysis was performed by matching EVAR to OAR patients controlling for age, gender and concomitant diseases. P values of less than 0.05 were considered significant. The statistical analysis of the data was performed using R (R version 3.4.1, R-core Team, R Foundation for Statistical Computing, Vienna, Austria, 2017, https://www.r-project.org), graphs with “survminer” and propensity score analysis with "MatchIt" R packages.

The study was not considered for review by the local ethical committee since the database was previously collected by a government agency and all data were fully anonymized, and fully encrypted before the authors had any access to them. Moreover, there was no direct patient contact whatsoever.

## Results

A total of 7 805 patients underwent repair of AAA using open or endovascular method and were followed for a median of 27.5 months (IQR 10.0–38.4 months). EVAR was performed in 5469 of patients (70.1%) who were significantly older, with a higher incidence of concomitant disorders, as compared with 2336 patients treated with OAR. The demographic data are presented in Table 1, percentage of EVAR in consecutive years of study on S1 Fig.

**Table 1:** Patient demographic data for entire population, age groups and unadjusted comparison by repair type and age group.

	All patients	OAR	EVAR	p	All 70+	OAR 70+	EVAR 70+	p	All 70-	OAR 70-	EVAR 70-	p	p 70+ vs 70- ^c^
n (%)	7805 (100)	2336 (29.9)^a^	5469 (70.1)^a^		4232 (54.2)^a^	1016 (24.0)^b^	3216 (76.0)^b^		3573 (45.8)^a^	1320 (36.9)^b^	2253 (63.1)^b^		<0.001
Age (yrs.)	70.9±8.1	68.5±7.7	72.0±8.1	< 0.001	77.1±4.7	75.6 ± 4.1	77.6±4.8	< 0.001	63.7±4.6	63.0±4.7	64.1±4.5	< 0.001	<0.001
Males	6693 (85.8)	1982 (84.8)	4711 (86.1)	0.138	3533 (83.5)	832 (81.9)	2701(84.0)	0.121	3160 (88.4)	1150 (87.1)	2010 (89.2)	0.065	<0.001
HTN	4104 (52.6)	1139 (48.8)	2965 (54.2)	< 0.001	2260 (53.4)	520 (51.2)	1740 (54.1)	0.105	1844 (51.6)	619 (46.9)	1225 (54.4)	< 0.001	0.119
CRF	289 (3.7)	64 (2.7)	225 (4.1)	0.003	186 (4.4)	40 (3.9)	146 (4.5)	0.482	103 (2.9)	24 (1.8)	79 (3.5)	0.004	<0.001
DM	1120 (14.3)	265 (11.3)	855 (15.6)	< 0.001	603 (14.2)	126 (12.4)	477 (14.8)	0.057	517 (14.5)	139 (10.5)	378 (16.8)	< 0.001	0.806
CAD	1059 (13.6)	222 (9.5)	837 (15.3)	< 0.001	565 (13.4)	95 (9.4)	470 (14.6)	< 0.001	494 (13.8)	127 (9.6)	367 (16.3)	< 0.001	0.564
Stroke	241 (3.1)	45 (1.9)	196 (3.6)	< 0.001	136 (3.2)	24 (2.4)	112 (3.5)	0.083	105 (2.9)	21 (1.6)	84 (3.7)	< 0.001	0.526

HTN—hypertension, CRF—chronic renal failure, DM—diabetes mellitus, CAD—coronary artery disease. Data presented as means±SD or numbers (percentages).

^a^ percentages were calculated vs. all patients,

^b^ percentages were calculated vs. all 70+ patients or all 70- patients respectively,

^c^ for comparison of all 70+ patients vs. all 70- patients

### Short-term mortality and long-term survival

Short-term (30-day mortality) among OAR group was significantly higher as compared to EVAR group (respectively, 0.5% vs. 0.2%, P = 0.035 for crude mortality). EVAR patients had higher long-term all-cause mortality (respectively 6.4% vs. 4.6%, P = 0.002 for crude mortality). Thirty-day and long-term mortality in all patients are presented in Table 2.

**Table 2:** Mortality and readmissions according to procedure and age group.

	All	OAR	EVAR	p	All 70+	OAR 70+	EVAR 70+	p	All 70-	OAR 70-	EVAR 70-	p	p 70+ vs 70-
n	7805	2336	5469		4232	1016	3216		3573	1320	2253		
All cause mortality	458 (5.9)	108 (4.6)	350 (6.4)	0.002	310 (7.3)	66 (6.5)	244 (7.6)	0.269	148 (4.1)	42 (2.2)	106 (4.7)	0.029	0.003
30-day all-cause mortality	23 (0.3)	12 (0.5)	11 (0.2)	0.037	16 (0.4)	8 (0.8)	8 (0.2)	0.033	7 (0.2)	4 (0.3)	3 (0.1)	0.435	0.035
Readmissions [number of pts.]	1098 (14.1)	195 (8.4)	903 (16.5)	<0.001	586 (13.8)	86 (8.5)	500 (15.5)	<0.001	512 (14.3)	109 (8.3)	403 (17.9)	<0.001	<0.001

Data presented as numbers with percentages in parentheses.

**Fig 1:**
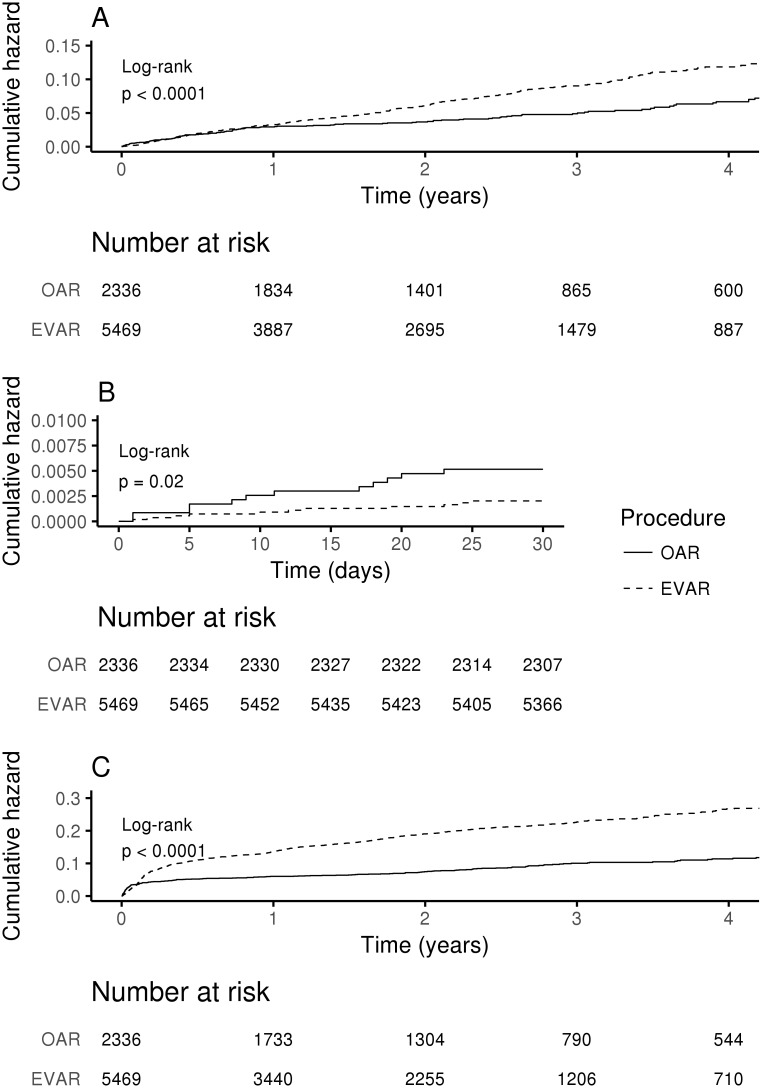
Kaplan-Meier analysis of mortality and readmissions by repair type. (A) Total mortality. (B) 30-day mortality. (C) Readmissions.

All-cause mortality trends over time are presented in Fig 1 with respective significant log-rank P values for comparison of Kaplan-Meier curves. The mortality curves diverge after 9.9 months. After this time EVAR subjects had a consistently higher mortality risk.

Cox analysis revealed that the type of procedure influenced both long-term and short-term survival independently (Tables 3 and 4). The analysis revealed also age as an independent covariant of long-term mortality (Table 3), and age and previous stroke for short-term mortality (Table 4).

**Table 3:** Long-term risk of death according to type of procedure and associated risk.

	All			70+			70-		
	HR	95% CI	P	HR	95% CI	P	HR	95% CI	P
EVAR vs OAR	1.34	1.07–1.67	0.010	1.18	0.89–1.56	0.244	1.59	1.10–2.30	0.014
Gender—female	1.00	0.77–1.30	0.984	0.98	0.72–1.34	0.917	1.06	0.65–1.74	0.819
Age	1.05	1.03–1.06	<0.001	1.06	1.04–1.09	<0.001	1.06	1.02–1.11	0.003
HTN	0.92	0.76–1.12	0.394	0.89	0.71–1.13	0.346	0.96	0.68–1.35	0.814
CRF	1.32	0.84–2.08	0.224	1.00	0.54–1.82	0.991	2.22	1.12–4.38	0.022
CAD	1.07	0.80–1.43	0.640	0.95	0.65–1.38	0.790	1.31	0.82–2.08	0.254
DM	0.94	0.71–1.29	0.683	0.99	0.70–1.41	0.961	0.84	0.52–1.38	0.500
Stroke	1.35	0.82–2.23	0.241	1.27	0.67–2.400	0.459	1.45	0.64–3.31	0.377
Readmission	1.14	1.00–1.30	0.059	1.15	0.97–1.36	0.113	1.13	0.90–1.42	0.278

HTN—hypertension, CRF—chronic renal failure, CAD—coronary artery disease, DM—diabetes mellitus

**Table 4:** Short term (30 days) risk of death according to type of procedure and associated risk.

	All			70+			70-		
	HR	95% CI	P	HR	95% CI	P	HR	95% CI	P
EVAR vs OAR	0.29	0.12–0.67	0.004	0.27	0.10–0.75	0.012	0.32	0.07–1.53	0.156
Gender—female	1.49	0.55–4.05	0.434	2.25	0.77–6.55	0.136	NA^a^		
Age	1.08	1.02–1.14	0.009	1.08	0.98–1.20	0.117	1.19	0.84–1.50	0.135
HTN	0.79	0.34–1.84	0.577	0.65	0.23–1.80	0.405	1.34	0.23–5.50	0.875
CRF	1.14	0.15–8.57	0.897	1.58	0.21–12.14	0.659	NA^a^		
CAD	1.17	0.34–4.08	0.803	0.53	0.07–4.15	0.547	2.75	0.50–15.16	0.245
DM	0.62	0.14–2.72	0.530	1.04	0.23–4.70	0.962	NA^a^		
Stroke	5.70	1.67–19.54	0.006	5.43	1.22–24.29	0.027	5.58	0.63–49.71	0.123

HTN—hypertension, CRF—chronic renal failure, CAD—coronary artery disease, DM—diabetes mellitus

^a^ calculations cannot be performed due to the zero incidence of female gender, previous hypertension and renal failure respectively among patients who died in 70- group.

**Fig 2:**
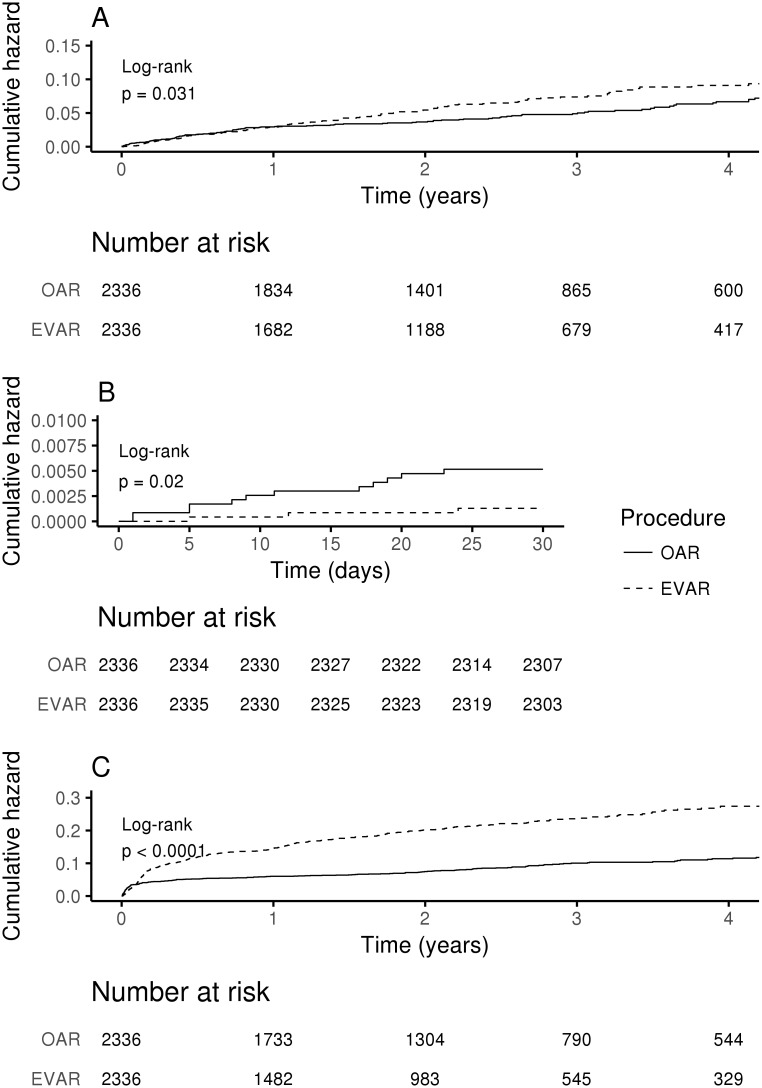
Kaplan-Meier analysis of mortality and readmissions by repair type in propensity score analysis. (A) Total mortality. (B) 30-day mortality. (C) Readmissions.

Kaplan-Meier curves for short and long-term mortality remained statistically different between OAR and EVAR patients after propensity score matching (Fig 2, S4 Table).

### Readmissions

**Fig 3:**
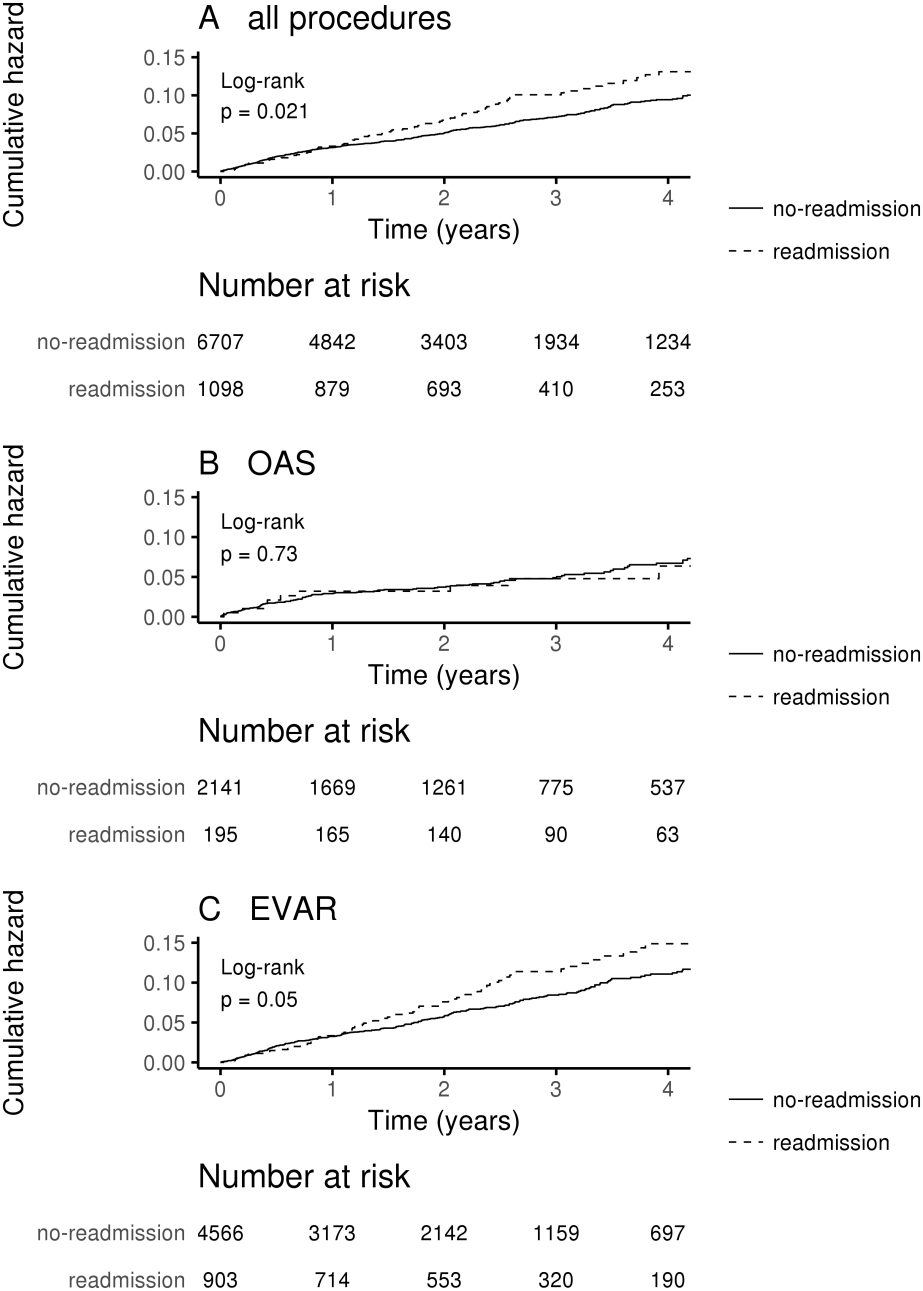
Kaplan-Meier analysis of long-term mortality according to readmission and repair type. (A) All procedures according to readmission. (B) OAR patients according to readmission. (C) EVAR patients according to readmission.

Readmissions occurred more frequently in EVAR than OAR group (16.5% vs. 8.3%, P<0.001 for crude data) (Table 2). According to Cox analysis readmissions were independently affected by the type of procedure (adjHR 2.15, 95%CI 1.84–2.52, P<0.001) and age (adjHR 0.99 95%CI 0.98–0.99, P = 0.001). Readmissions were associated with increased long-term mortality in EVAR, but not in OAR patients only in unadjusted analysis (Fig 3, Table 3). Propensity score analysis revealed significant differences in readmissions between OAR and EVAR (S4 Table, Fig 2).

### Sub-analysis in age subgroups

**Fig 4:**
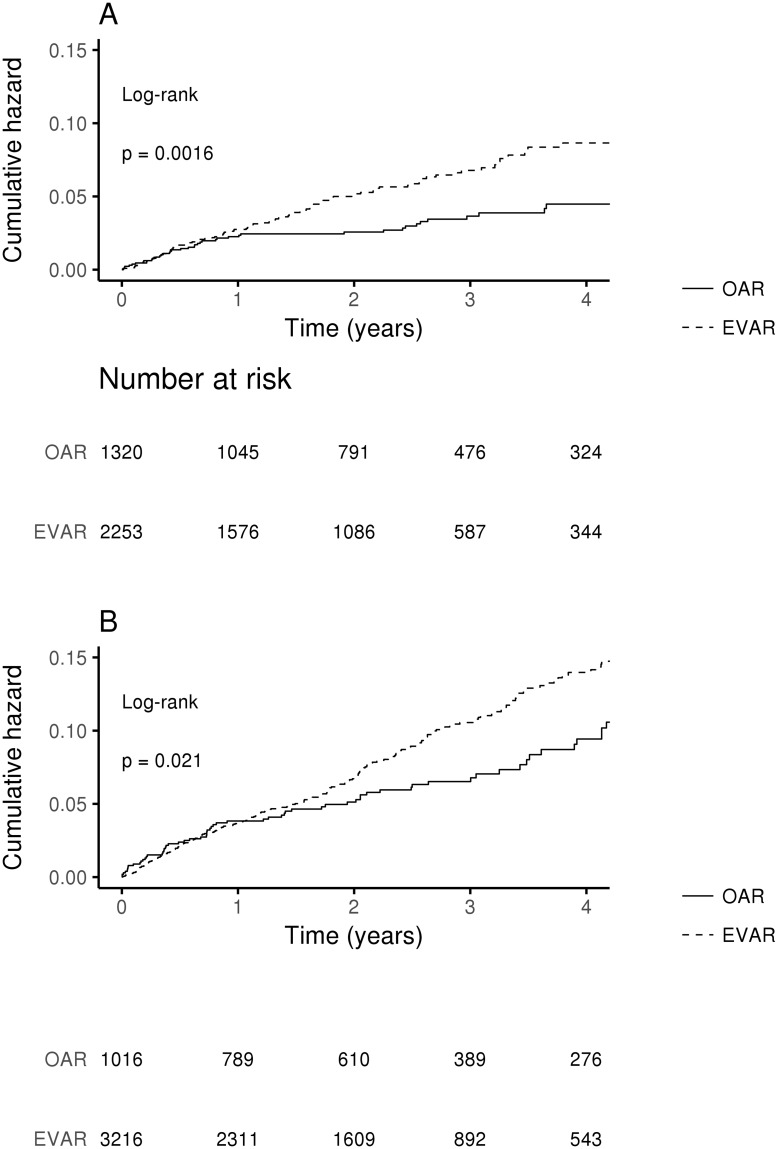
Kaplan-Meier analysis of long-term mortality according to the age group. (A) Long-term mortality in 70- patients. (B) Long-term mortality in 70+ patients.

Comparison of demographic data between 70+ and 70- patients as well as between EVAR and OAR in each age subgroup separately is presented in Table 1. Short and long term all-cause unadjusted crude mortality was higher in the 70+ group than 70- (Table 2). Short-term crude mortality was higher in OAR vs. EVAR in 70+ group, but no difference was seen in 70- group. Long-term crude mortality was higher in EVAR vs. OAR only in 70- but not in 70+ group (Table 2). Kaplan-Meier analysis revealed higher unadjusted long-term mortality in EVAR vs. OAR patients in 70+ as well as in 70- groups (Fig 4).

In adjusted, Cox analysis the type of procedure significantly affected all-cause long-term mortality only in the 70- group (Table 3), but 30-day all-cause mortality only in the 70+ group (Table 4). Age was a significant covariant in both age groups regarding long term-mortality, but regarding 30-day mortality only in the 70+ group (Tables 3 and 4). Renal failure significantly increased the risk of long-term mortality in the 70- group, while previous stroke markedly increased the risk of 30-day mortality in the 70+ group (Tables 3 and 4).

## Discussion

Our analysis of 7805 patients treated for AAA with a median follow up 27.5 months revealed survival benefit of EVAR over OAR disappeared early during the first year after the procedure.

In randomized trials and observational studies both in-hospital and 30-day mortality was significantly higher in OAR than EVAR patients [5,6,9], and this observation was confirmed in our study in all patients and in the 70+ group. It should be emphasized, that very low short-term mortality after AAA repair observed in our analysis in contrast to many earlier reports was also described in some recent studies. DREAM study reported the 30-day mortality after EVAR as 0.4% [12]. In Swedvasc registry patients with screening-detected AAA had 1.0% mortality after surgery and 0% after EVAR [13]. Age was an independent risk covariant of short-term mortality in all patients and in 70+ group. Although results of many studies indicated, that the essential predictor of vascular complications after EVAR was difficult anatomy of the abdominal aorta rather than co-morbidity [14,15], the new and previously unreported finding of our study was a very strong influence of the history of stroke on short-term mortality that may be explained by more advanced atherosclerosis or higher frequency of atrial fibrillation.

Recent meta-analyses of randomized trials [16,17] showed that after four years, early advantage of EVAR disappeared, mostly due to aneurysm-related mortality. Age, the presence of coronary heart disease, diabetes or chronic kidney disease did not affect survival, but the presence of PAD was associated with increased mortality in EVAR group [17]. Follow-up of EVAR-1 patients for 15 years confirmed increased late mortality in EVAR group due to higher incidence of aneurysmal complications and cancer [18].

Observational studies may have some advantages as compared with randomized trials, since they represent current, every-day practice and better reflects the patients population allocated for different types of treatment [6]. In our study we described an approximately 50% higher long term mortality in EVAR than in OAR patients (6.4 vs. 4.6%), that could be seen after 10 months of follow-up. Analyses reporting long-term survival and outcomes of EVAR and OAR on a population level comparable with our study are scarce [7,8]. The largest long-term observational study by Chang et al. revealed, that after 3 years EVAR repair was associated with non-significant higher mortality compared with OAR [8]. Another retrospective analysis of 4 529 patients showed an increased risk of total and AAA-related mortality during the entire post-operative period in subjects treated with OAR [7].

Lower long-term survival rates in randomized and earlier observational studies, as compared to our cohort, may be explained by younger age of our studied population [5,6,8,16,18], however the differences in Kaplan-Meier estimator remained significant after propensity score matching. Our data demonstrate low-perioperative risk in the study group despite the fact, that prevalence of hypertension, diabetes mellitus, chronic kidney disease, coronary artery disease and the history of stroke as well as proportion of female patients were similar to other studies which reported relevant data [7,8,17].

Also, it may be speculated, that lower short-term and long-term mortality in our group may result from the differences in aneurysmal diameters and referral of many patients with small AAA [9] with less complex anatomy, use of latest generations of stengrafts [19].

International guidelines recommend that intervention should be considered once the aneurysm diameter exceeds 55 mm in men or 50 mm in women [4], but the proportion of aneurysms that are repaired at a diameter of less than 55 mm has been reported to range from 6.4 to 29.0% in various countries [9] and Polish guidelines since 2009 suggest 5 mm lower thresholds for EVAR [20].

Predicting late survival before elective AAA repair remains the Achilles’ heel of AAA management [21]. Randomized studies which use strict inclusion and exclusion criteria may not identify some risk factors influencing long-term mortality, that may be important in real world in patients undergoing AAA repair.

The recent systematic review and meta-analysis of relevant articles reporting risk factors influencing long-term survival following OAR and EVAR revealed that patients with end stage renal disease and advanced COPD had the worst long-term survival with HR 3.15 and HR 3.05 for death, respectively [21]. An increase in age was associated with HR of 1.05 similarly as in our study, but we did not find worse survival in women as compared to men. Neither comorbidity tested in our analysis using preprocedural ICD-10 codes did not reveal significant influence on long-term morality in the whole group, although HR values for chronic renal failure and previous stroke were similar to the above mentioned meta-analysis. Chronic renal failure was linked to worse survival in 70- group.

Similar to other studies [16], repeated hospitalizations were more frequent in EVAR than OAR groups (16.5% vs 8.4%, p<0.001) independently of other risk factors, except age. Due to the fact, that re-interventions may be coded in various ways and may result from concomitant diseases we decided to include in our analysis readmissions to vascular units as a measure of late complications requiring interventions.

### Limitations of the study

Our study presents the typical limitations of a retrospective analysis of reimbursement data. However, the data were collected systematically and prospectively by the single insurer all over the country. Due to the limitations of the NHF database we were unable to assess many other important parameters such as smoking, prevalence of COPD or PAD, preoperative physical status, AAA diameter and anatomy, type of endovascular device. Since a majority of deaths occurred outside hospitals and autopsies are rarely performed in Poland we were unable to establish the cause of death. There is a risk of potential errors or underreporting of certain diagnoses or procedures. Non-randomized design is another obvious limitation.

## Conclusions

Our large-population based study of patients treated for unruptured AAA revealed significantly higher late mortality after EVAR than after OAR, particularly in patients below 70 years of age. Compared to other studies we have found lower short-term and long-term mortality in our cohort, which suggests lower cardiovascular risk in treated population. However, contrary to other studies, survival benefit of EVAR disappeared early during the first year after the procedure, accompanied by an increased frequency of readmissions of EVAR patients. Taking into consideration significant variations in the management of AAA, in particular low compliance with EVAR device guidelines [1] our data necessitate re-evaluation of the strategy for AAA management in vascular units in the country.

## Supporting information

S1 Fig Relative frequency of EVAR procedures by year. (PDF)

S1 Table Inclusion/exclusion criteria according to International Classification of Diseases, Ninth Revision (ICD-9) for procedures and International Classification of Diseases, Tenth Revision (ICD-10) for diagnosis. AAA—abdominal aortic aneurysm.
(DOC)

S2 Table International Classification of Diseases, Tenth Revision codes for concomitant diseases. (DOC)

S3 Table Kaplan-Maier survival function estimator in consecutive years for mortality and readmissions according to procedure with 95% confidence intervals in brackets. (DOC)

S4 Table Patient demographic and end-point data for OAR and propensity score matched EVAR patients. HTN—hypertension, CRF—chronic renal failure, DM—diabetes mellitus, CAD—coronary artery disease. Data presented as means±SD or numbers (percentages).
(DOC)
