# Supplementary figures and images for: Short- and long-term survival after open versus endovascular repair of abdominal aortic aneurysm—Polish population analysis
Source: PLoS One. 2018 Jun 14;13(6):e0198966. doi: 10.1371/journal.pone.0198966 (PMC6002078; doi:10.1371/journal.pone.0198966)

**Percentage of EVAR procedures**

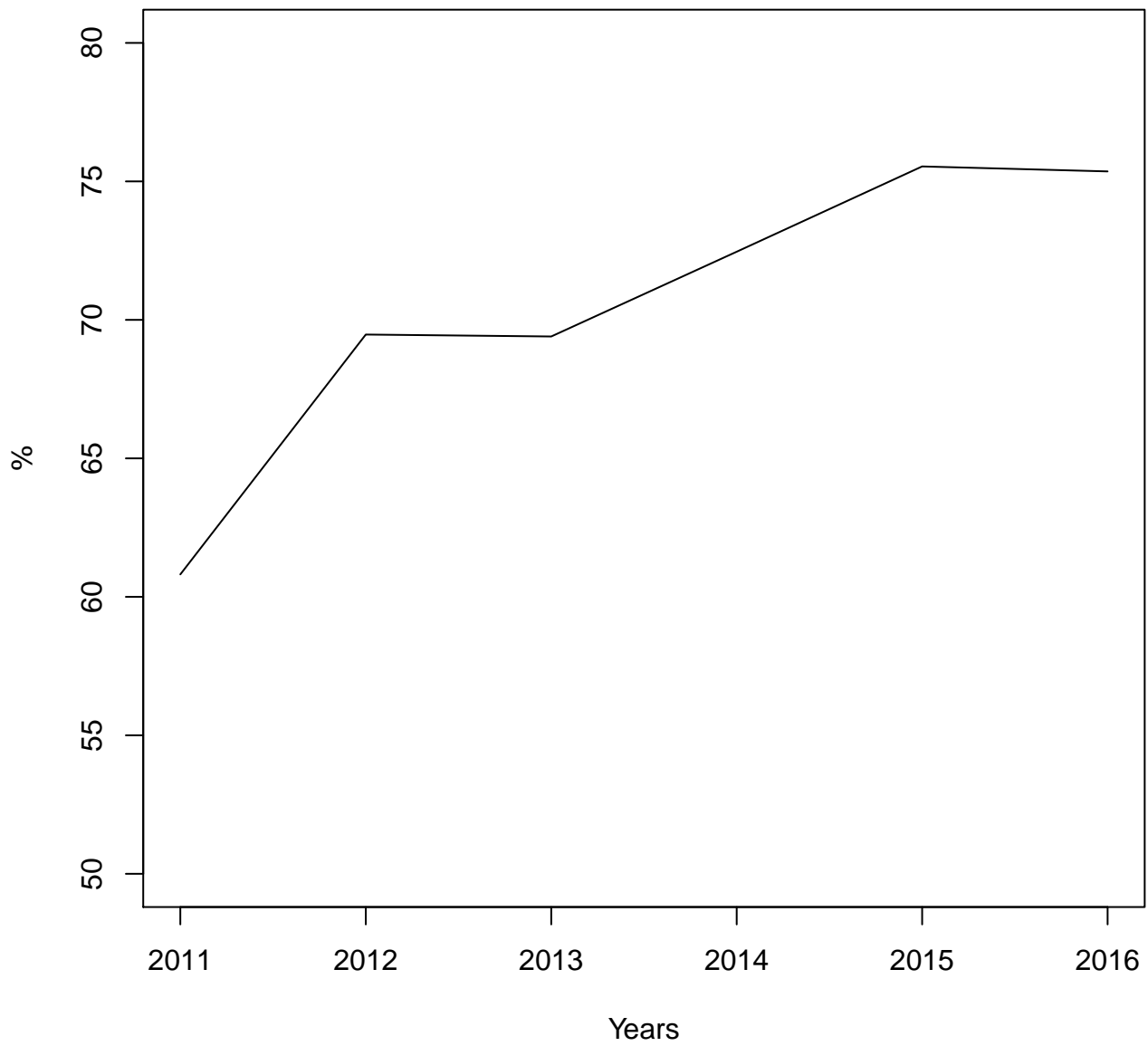

Supplement: S1 Fig — (PDF) [file pone.0198966.s001.pdf]
