# Supplementary material for: Short- and long-term survival after open versus endovascular repair of abdominal aortic aneurysm—Polish population analysis
Source: PLoS One. 2018 Jun 14;13(6):e0198966. doi: 10.1371/journal.pone.0198966 (PMC6002078; doi:10.1371/journal.pone.0198966)
Supplement: S1 Table — AAA—abdominal aortic aneurysm. (DOC) [file pone.0198966.s002.doc]

| **Inclusion criteria:** |
| --- |
| Unruptured AAA: I71.4 |
| treated with: |
| Open aortic surgery: 38.424 or |
| Endovascular repair of AAA with stent graft: 39.711 |
| **Exclusion criteria:** |
| Ruptured AAA: I71.3, I71.5 |
| AAA extended to thoracic artery: I71.5, I71.6 |
| AAA extended to iliac artery: I72.3 |
| treatment by other procedures than 38.424 or 39.711 |
