# Supplementary material for: Short- and long-term survival after open versus endovascular repair of abdominal aortic aneurysm—Polish population analysis
Source: PLoS One. 2018 Jun 14;13(6):e0198966. doi: 10.1371/journal.pone.0198966 (PMC6002078; doi:10.1371/journal.pone.0198966)
Supplement: S2 Table — (DOC) [file pone.0198966.s003.doc]

| Hypertension: I10-I15 |
| --- |
| Diabetes mellitus: E10-E14 |
| Coronary artery disease: I20-I24 |
| Chronic kidney disease: N18 |
| Stroke: I60-I64 |
