# Supplementary material for: Short- and long-term survival after open versus endovascular repair of abdominal aortic aneurysm—Polish population analysis
Source: PLoS One. 2018 Jun 14;13(6):e0198966. doi: 10.1371/journal.pone.0198966 (PMC6002078; doi:10.1371/journal.pone.0198966)
Supplement: S3 Table — (DOC) [file pone.0198966.s004.doc]

| Long-term mortality | OAS |  |  |  | EVAR |  |  |  |  |
| --- | --- | --- | --- | --- | --- | --- | --- | --- | --- |
| time | at risk | deaths | survival | (95%CI) | at risk | deaths | survival |  | (95%CI) |
| Baseline | 2336 | 0 | 1.000 | NA | 5469 | 0 | 1.000 |  | NA |
| 1 y | 1834 | 62 | 0.971 | (0.964-0.978) | 3887 | 152 | 0.968 |  | (0.963-0.973) |
| 2 y | 1401 | 12 | 0.964 | (0.956-0.972) | 2695 | 92 | 0.941 |  | (0.933-0.948) |
| 3 y | 865 | 15 | 0.952 | (0.942-0.962) | 1479 | 66 | 0.914 |  | (0.904-0.923) |
| 4 y | 600 | 12 | 0.935 | (0.922-0.949) | 887 | 30 | 0.888 |  | (0.875-0.901) |
| 5 y | 100 | 6 | 0.924 | (0.907-0.940) | 157 | 11 | 0.869 |  | (0.851-0.887) |
| Readmisions | OAS |  |  |  | EVAR |  |  |  |  |
| time | at risk | events | survival | (95%CI) | at risk | events | survival |  | (95%CI) |
| baseline. | 2336 | 0 | 1.000 | NA | 5469 | 0 | 1.000 |  | NA |
| 1 y | 1733 | 131 | 0.942 | (0.932-0.951) | 3440 | 636 | 0.873 |  | (0.864-0.882) |
| 2 y | 1304 | 21 | 0.928 | (0.917-0.939) | 2255 | 154 | 0.827 |  | (0.815-0.838) |
| 3 y | 790 | 28 | 0.904 | (0.891-0.919) | 1206 | 66 | 0.797 |  | (0.784-0.811) |
| 4 y | 544 | 8 | 0.892 | (0.876-0.909) | 710 | 34 | 0.766 |  | (0.749-0.782) |
| 5 y | 90 | 7 | 0.868 | (0.841-0.895) | 125 | 12 | 0.741 |  | (0.720-0.763) |
