# Supplementary material for: Short- and long-term survival after open versus endovascular repair of abdominal aortic aneurysm—Polish population analysis
Source: PLoS One. 2018 Jun 14;13(6):e0198966. doi: 10.1371/journal.pone.0198966 (PMC6002078; doi:10.1371/journal.pone.0198966)
Supplement: S4 Table — HTN—hypertension, CRF—chronic renal failure, DM—diabetes mellitus, CAD—coronary artery disease. Data presented as means±SD or numbers (percentages). (DOC) [file pone.0198966.s005.doc]

|  | OAR | EVAR | P |
| --- | --- | --- | --- |
| n (%) | 2336 | 2336 |  |
| Age (yrs.) | 68.5±7.7 | 68.7±8.0 | 0.289 |
| Males | 1982 (84.8) | 1993 (85.3) | 0.681 |
| HTN | 1139 (48.8) | 1137 (48.7) | 0.977 |
| CRF | 64 (2.7) | 62 (2.7) | 0.928 |
| DM | 265 (11.3) | 241 (10.3) | 0.279 |
| CAD | 222 (9.5) | 200 (9.1) | 0.284 |
| Stroke | 45 (1.9) | 38 (1.6) | 0.578 |
| All cause mortality | 108 (4.6) | 126 (5.4) | 0.226 |
| 30-day all-cause mortality | 12 (0.5) | 4 (0.2) | 0.076 |
| Readmissions  [number of pts.] | 195 (8.4) | 412 (17.6) | <0.001 |
